# Clinical Assessment of Endothelial Function in Convalescent COVID-19 Patients Undergoing Multidisciplinary Pulmonary Rehabilitation

**DOI:** 10.3390/biomedicines9060614

**Published:** 2021-05-28

**Authors:** Pasquale Ambrosino, Antonio Molino, Ilenia Calcaterra, Roberto Formisano, Silvia Stufano, Giorgio Alfredo Spedicato, Andrea Motta, Antimo Papa, Matteo Nicola Dario Di Minno, Mauro Maniscalco

**Affiliations:** 1 Istituti Clinici Scientifici Maugeri IRCCS, 27100 Pavia, Italy; pasquale.ambrosino@icsmaugeri.it (P.A.); roberto.formisano@icsmaugeri.it (R.F.); silvia.stufano@icsmaugeri.it (S.S.); antimo.papa@icsmaugeri.it (A.P.); 2 Department of Respiratory Medicine, Federico II University, 80131 Naples, Italy; molinotonio@libero.it; 3 Department of Clinical Medicine and Surgery, Federico II University, 80131 Naples, Italy; ileniacalcaterra@hotmail.it; 4 Department of Data Analytics and Actuarial Science, Unipol Group, 40128 Bologna, Italy; spedicato_giorgio@yahoo.it; 5 Institute of Biomolecular Chemistry, National Research Council, ICB-CNR, 80078 Pozzuoli, Naples, Italy; andrea.motta@icb.cnr.it; 6 Department of Translational Medical Sciences, Federico II University, 80131 Naples, Italy

**Keywords:** COVID-19, biomarkers, endothelial function, rehabilitation, disability, exercise, outcomes

## Abstract

Background: Growing evidence points to a key role of endothelial dysfunction in the pathogenesis of COVID-19. In this study, we evaluated changes in endothelium-dependent flow-mediated dilation (FMD) in a cohort of convalescent COVID-19 patients undergoing pulmonary rehabilitation (PR). Methods: After swab test negativization, convalescent COVID-19 patients referring to a post-acute care facility for PR were consecutively screened for inclusion. Study procedures were performed at the time of hospitalization and discharge. Results: We enrolled 82 convalescent COVID-19 patients (85.4% males, mean age 60.4 years). After PR, a significant improvement in most pulmonary function tests and exercise capacity was documented. FMD changed from 2.48% ± 2.01 to 4.24% ± 2.81 (*p* < 0.001), corresponding to a 70.9% increase. Significantly higher changes in FMD were found in patients without a history of vascular events as compared to those with (+2.04% ± 2.30 vs. +0.61% ± 1.83, *p* = 0.013). Values of forced expiratory volume in 1 s (FEV_1_%), forced vital capacity (FVC%) and diffusion capacity for carbon monoxide (DLCO%) significantly and directly correlated with FMD both at baseline and after PR. Patients with normal FEV_1_% (≥80% predicted) during the overall study period or those normalizing FEV_1_% after PR showed a more significant FMD change as compared to patients with persistently impaired FEV_1_% (<80% predicted) (*p* for trend = 0.029). This finding was confirmed in a multivariate analysis. Conclusions: Clinically evaluated endothelial function improves after PR in convalescent COVID-19 patients. A direct and persistent association between the severity of pulmonary and vascular disease can be hypothesized. Endothelial function testing may be useful in the follow-up of convalescent COVID-19 patients.

## 1. Introduction

In December 2019, the novel severe acute respiratory syndrome coronavirus 2 (SARS-CoV-2) first appeared in Hubei Province, China [1]. In spite of the efforts to contrast its diffusion, SARS-CoV-2 has been responsible for a global health emergency [2], culminating in the World Health Organization (WHO) pandemic declaration in March 2020 [3]. Epidemiologic data on SARS-CoV-2 infection reveal that the viral agent can cause coronavirus disease 2019 (COVID-19) [4], a condition with a wide spectrum of clinical manifestations, ranging from an asymptomatic disease to a critical condition requiring intensive care unit (ICU) management [5].

Reports of persistent clinical manifestations in patients recovering from COVID-19 are emerging [6], thus suggesting the presence of a “post-COVID-19 syndrome” [7] and the need for post-acute pulmonary rehabilitation (PR) after swab test negativization [8]. This is consistent with the finding of both radiological and functional pulmonary abnormalities in a substantial proportion of COVID-19 survivors at hospital discharge and even months after discharge [9,10]. In keeping with this, evidence of long-term cardiovascular (CV) consequences which are independent from pre-existing conditions and disease severity has been documented in several reports [11,12]. However, the mechanisms of the post-acute manifestations of COVID-19 are still under investigation [13].

The accumulated evidence points to the key role of endothelial dysfunction in the pathogenesis of most COVID-19 manifestations [14], since vascular endothelial cells can be infected by SARS-CoV-2 with subsequent endothelial injury [15]. Moreover, it has been shown that the residual activation of the immune system is responsible for persistent endothelial dysfunction even after the acute phase [16]. This has led the European Society of Cardiology to recommend the clinical assessment of endothelial function in the follow-up of all convalescent COVID-19 patients for early detection of long-term CV complications [17].

To date, several laboratory and clinical tests have been proposed to measure endothelial function in humans [18]. Among them, flow-mediated dilation (FMD) is recognized as a non-invasive and accurate clinical method [19] and as a surrogate marker of subclinical atherosclerosis [20], with a good responsiveness to interventions and correlation with coronary endothelial function [21]. Moreover, FMD is currently considered an independent predictor of CV events [22], thus providing important prognostic data over and above traditional CV risk factors.

The aim of our study was to prospectively assess the changes in endothelium-dependent FMD in a cohort of convalescent COVID-19 patients undergoing in-hospital PR after swab test negativization.

## 2. Materials and Methods

### 2.1. Methods

Convalescent COVID-19 patients referring to the Pulmonary Rehabilitation Unit of Istituti Clinici Scientifici Maugeri Spa SB, IRCCS of Telese Terme, Benevento, Italy, after swab test negativization were consecutively screened for enrolment between December 2020 and March 2021. The major inclusion criteria were: previous SARS-CoV-2 infection, confirmed by reverse transcription polymerase chain reaction (RT-PCR) of the nasopharyngeal swab; history of severe or critical COVID-19 according to World Health Organization (WHO) classification; negativization of two nasopharyngeal swab tests for SARS-CoV-2 (spaced 1 week apart) within the last 2 months; indication for in-hospital PR due to persistent clinical manifestations of COVID-19 after the acute phase. Exclusion criteria were: age <18 years; any previous surgical procedure of the lungs; active malignant disease (except basal cell carcinoma of the skin); acute myocardial infarction or stroke within the last 6 months; any major surgical procedure during the 6 months prior to enrolment; inability to understand relevant information related to the study protocol and to sign informed consent; history of alcohol or drug abuse or any other condition associated with poor compliance with the study procedures. Patients with missing data for the outcome of interest were excluded from the study.

This study followed the Strengthening the Reporting of Observational Studies in Epidemiology (STROBE) reporting guidelines [23] and was conducted in accordance with the Declaration of Helsinki of the World Medical Association. The Institutional Review Board of Istituto Nazionale Tumori, Fondazione Pascale, Naples, Italy, approved (16 December 2020) the study with reference number ICS 11/20. Written informed consent was obtained from all subjects involved in the study.

### 2.2. Study Protocol

After signing the informed consent, the major demographic and clinical information relating to pre-existing and current medical conditions as well as ongoing treatments was collected for all included patients.

The presence of CV risk factors was established according to the National Cholesterol Education Program (NCEP) criteria [24]. In detail, abdominal obesity was defined by a waist circumference ≥88 cm for women and ≥102 cm for men, hypertriglyceridemia by triglycerides levels ≥150 mg/dL, hypercholesterolemia by total cholesterol ≥200 mg/dL regardless of the presence of high-density lipoprotein (HDL) cholesterol <50 mg/dL for women and <40 mg/dL for men, hypertension by a systolic blood pressure (SBP) ≥130 mmHg and/or a diastolic blood pressure (DBP) ≥85 mmHg, and impaired fasting glucose (IFG) by fasting glucose ≥100 mg/dL.

All study procedures were performed in a room with a controlled temperature of 23 degrees Celsius. Each measurement was obtained at admission and repeated at discharge, following the PR program conclusion.

### 2.3. Study Procedures

The main anthropometric, clinical and laboratory parameters were measured in each patient. Body weight was assessed after bladder emptying, with the subject barefoot and in light clothing. A digital scale with an accuracy of 0.1 kg was used. Height was measured to the nearest 0.1 cm. Body mass index (BMI) was calculated as body weight/height^2^.

SBP and DBP were measured in a lying position on three occasions (spaced 1 min apart) according to European Society of Hypertension/European Society of Cardiology protocols [25]. The mean value of the three measurements was calculated for each patient.

We collected a venous blood sample from all participants in fasting conditions. Glucose, creatinine, total cholesterol, triglycerides, urea, uric acid, transaminases, C-reactive protein (CRP), D-Dimer, and complete blood count (CBC) parameters were measured locally in all study subjects.

Arterial blood gas [oxygen (PaO_2_) and carbon dioxide tension (PaCO_2_)] and the power of hydrogen (pH) and bicarbonate concentrations (HCO_3_^−^) were evaluated with a blood gas analyzer (ABL 825^®^ FLEX BGA, Radiometer Medical Aps, Copenhagen, Denmark). All measurements followed the European Respiratory Society recommendations [26].

Forced expiratory volume in 1 s (FEV_1_) and forced vital capacity (FVC) were measured using an automated instrument (Vmax^®^ Encore, Vyasis Healthcare, Milan, Italy), following the American Thoracic Society/European Respiratory Society (ATS/ERS) protocols [27,28]. Single-breath lung diffusion capacity for carbon monoxide (DLCO) was measured according to the ATS/ERS recommendations, resulting from the product of the carbon monoxide transfer coefficient (KCO) multiplied by alveolar volume (VA) [29]. FEV_1_, FVC, DLCO and DLCO/VA were expressed both as absolute values and percentages of predicted values (FEV_1_%, FVC%, DLCO% and DLCO/VA%, respectively).

Although specifically designed for COPD patients, the COPD Assessment Test (CAT) was used to evaluate health status impairment in our population [30]. The Barthel score was also calculated to determine the level of functioning and to monitor improvements in activities of daily living over time [31].

In agreement with reported protocols [32], functional exercise capacity was expressed in meters (m) with the six-minute walking distance (6MWD).

### 2.4. Brachial Artery Flow-Mediated Dilation (FMD)

We asked patients to refrain from caffeine, tobacco and alcohol for at least 12 h before the test. Study protocols were carried out after overnight fasting and after at least 10 min of supine rest, accepting a small head pillow if requested by the patient. All examinations were performed by the same operator, who was unaware of the clinical status of the patients.

The test consisted of measuring brachial artery diameter (BAD) at rest and after reactive hyperemia (RH) caused by forearm ischemia, following the protocol of the International Brachial Artery Reactivity Task Force [19]. BAD was measured on an ultrasound section of the artery in B-mode, visualized above the cubital fossa in the longitudinal plane using a 10 MHz linear probe and a Vivid E95^®^ (GE Healthcare, Chicago, IL, USA) ultrasound instrument. Baseline BAD and velocity of the blood flow were recorded for the first 60 s. The blood pressure cuff was placed on the forearm 4–5 cm below the elbow crease and inflated to 70 mmHg above SBP in order to determine a transient ischemia of 300 s. After 300 s, the cuff was deflated recording the BAD and the flow velocity for the next 240 s. A software for the analysis of ultrasound images (Cardiovascular Suite^®^, FMD studio, QUIPU Srl, Pisa, Italy) was used, permitting an automatic and real-time calculation of the vascular reactivity parameters.

FMD was calculated as [(maximum post-ischemic BAD—basal BAD)/basal BAD] × 100 and expressed as percent (%) of BAD increase compared with baseline value [19]. The shear stress stimulus to FMD was defined by the shear rate area under the curve from cuff deflation to peak diameter (SR_AUC_) [33], with shear rate being calculated as 4 × blood velocity/BAD [34]. Normalized FMD was calculated as (FMD/SR_AUC_) × 10^4^ (arbitrary units) to minimize individual variability in the hyperemic stimulus [35]. The full post-occlusive RH during 240 s after deflation was defined by total share rate area under the curve (SR_AUC-TOT_) as well as by the ratio of average flow velocity after cuff deflation/flow velocity measured at baseline [36,37]. Considering the initial rest period and preliminary procedures, the entire exam lasted an average of 20 min per patient. We evaluated the protocol reproducibility on a test set of 5 patients randomly chosen from the study population within 1 week from the start of the study.

### 2.5. Pulmonary Rehabilitation

The interdisciplinary PR program delivered to patients with respiratory diseases in our unit is in agreement with the key concepts of the official ATS/ERS recommendations [38]. In brief, after an accurate evaluation of all pulmonary and extra-pulmonary conditions, each patient underwent a rehabilitation intervention with a multidisciplinary and individualized approach, based on physical exercise, psychological support, nutritional counselling and occupational therapy. Exercise training was the mainstay of the program. Upper and lower limb strengthening exercises were performed using body weight as well as free and fixed weights at a load that could be supported for 8–10 repetitions before muscle exhaustion. For each exercise, loads were increased if participants were able to complete, without muscle exhaustion, at least three sets of 8–10 repetitions in two consecutive sessions. Arm ergometry, treadmill walking and/or stationary cycling were performed for 30 min per session, aimed at scoring dyspnea or perceived exertion from 3 to 4 on a 0 to 10 category-ratio scale [39,40]. All patients also underwent flexibility and stretching exercises. Participation was monitored and supervised by physiotherapists.

### 2.6. Statistical Analysis

Statistical analysis was carried out with R Statistical software (R Core Team 2021). Continuous data were expressed as mean ± standard deviation (SD). Categorical variables were summarized as relative frequencies. To compare continuous variables, we resorted to the paired samples *t*-test. The Mann–Whitney U test was used to compare means in case of values presenting with a skewed non-Gaussian distribution. A repeated measures analysis of variance (ANOVA) with mixed effects modelling was performed to evaluate the interaction of categorical variables with time and associated changes in FMD over time. Relationships between continuous variables and changes in FMD during the study period were examined using Spearman’s correlation coefficient (rho). We used a linear regression analysis (stepwise method) to adjust for potential confounders and to identify predictors. A *p* < 0.05 was considered statically significant.

All analyses were repeated after stratifying patients according to the presence of any vascular complication (at least one among stroke, transient ischemic attack, cardiac events, peripheral artery disease, venous thromboembolism), any traditional CV risk factor (at least one among obesity, hypertriglyceridemia, hypercholesterolemia, hypertension, IFG, diabetes mellitus, smoking) and the other major clinical and demographic characteristics.

### 2.7. Sample Size

Based on data of a recent study on a similar patient population [41], a sample of 63 patients was considered necessary to detect an expected pre-defined increase in FMD from baseline values of ≥40% with a power of 80% and a level of significance of 5%. To account for possible drop-outs or technical failures during FMD measurements, we planned to screen ≈100 patients for eligibility.

## 3. Results

### 3.1. Subjects

As reported in Supplemental Figure S1, of 102 patients screened for eligibility, 14 (13.7%) were ineligible for protocol adherence issues. A total of 2 (2.3%) out of the 88 eligible patients were not considered because the baseline FMD measurement failed. A total of 86 patients, whose baseline measurements could be successfully assessed, started PR. Of these, three (3.5%) subjects withdrew from the study before completion of project requirements. One (1.2%) subject had an unsuccessful FMD measurement after PR.

Therefore, 82 convalescent COVID-19 patients (85.4% males, mean age 60.4 years) were considered for the final analysis. Table 1 reports the baseline characteristics of the enrolled patients. Information relating to ongoing pharmacological therapies and oxygen supplementation is summarized in Supplemental Table S1.

The study sample consisted of middle-aged patients with a recent history of severe (35.4%) or critical COVID-19 (64.6%). Most patients (68.3%) were transferred from an acute care setting after hospitalization with a mean length of stay of 23.2 days. A total of 23.2% of patients had been mechanically ventilated during the acute phase, while 26.8% had received high-flow oxygen therapy. Patients were generally overweight (mean BMI: 28.9), with 30.5% of the sample being obese subjects. About half of the patients had essential hypertension. However, considering the high percentage of subjects using antihypertensive agents, blood pressure values were generally normal at the time of FMD assessment. The prevalence of diabetes was 17.1%, and current smokers accounted for 9.8% of the study sample. A history of cardiac events and stroke was reported by 15.9% and 2.4%, respectively, whereas COPD was reported by 7.3% of the patients.

### 3.2. Changes in Main Laboratory and Functional Parameters

Changes in body composition, pulmonary function tests, laboratory parameters and other outcome measures in the 82 convalescent COVID-19 patients following the PR program conclusion are reported above (Table 1).

As compared to baseline, a significant increase in PaO_2_ and SpO_2_ as well as in PaCO_2_ values was documented (*p* always < 0.001). Moreover, an improvement in most spirometry parameters was reported at the end of the PR program, with FEV_1_% changing from 77.7% predicted ± 20.3 to 84.5% predicted ± 18.2 (*p* < 0.001) and FVC% from 74.9% predicted ± 18.9 to 82.1% predicted ± 16.4 (*p* < 0.001). In contrast, FEV_1_/FVC was substantially unchanged after PR (from 82.4% ± 8.7 to 81.6% ± 9.5, *p* = 0.241). Both DLCO% and DLCO/VA% significantly increased after PR, from 57.6% predicted ± 20.0 to 64.2% predicted ± 20.7 (*p* < 0.001) and from 85.2% predicted ± 18.6 to 89.7% predicted ± 15.0 (*p* = 0.004), respectively. A significant and consistent improvement in exercise capacity was also documented at the end of the PR program, with 6MWD changing from 194.9 ± 115.0 to 343.5 m ± 108.2 (*p* < 0.001). Similarly, self-assessment measures of health status impairment (CAT) and functional limitation (Barthel score) also significantly improved after PR (*p* always < 0.001).

### 3.3. Changes in FMD and Measures of Vascular Reactivity

After PR, FMD changed from 2.48% ± 2.01 to 4.24% ± 2.81 (*p* < 0.001), corresponding to a 70.9% increase. Similarly, a 77.6% increase in normalized FMD was also documented (from 1.34 ± 1.29 to 2.38 ± 2.27, *p* < 0.001). In contrast, no changes in BAD (from 4.02 ± 0.64 to 4.06 mm ± 0.65, *p* = 0.364), SR_AUC_ (from 26,401 ± 17,699 to 25,590 ± 18,680, *p* = 0.710), SR_AUC-TOT_ (from 54,745 ± 32,048 to 55,113 ± 35,176, *p* = 0.921), and RH (from 1.17 ± 0.16 to 1.19 ± 0.13, *p* = 0.372) were found after PR. 

**Figure 1:**
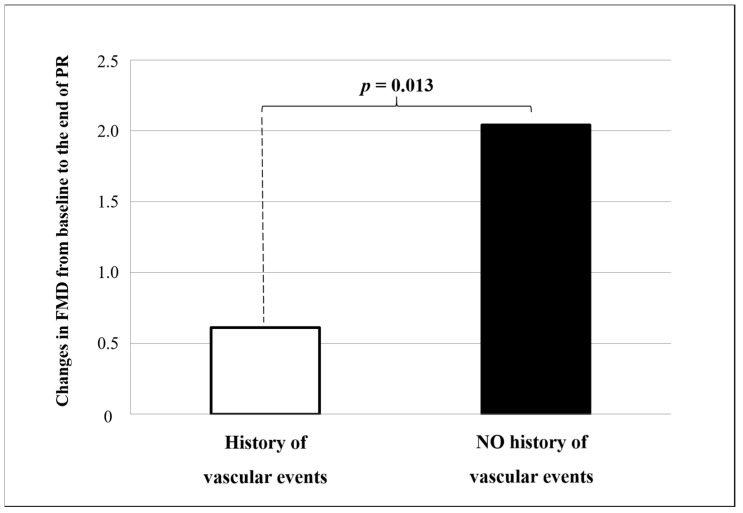
Changes in flow-mediated dilation (FMD) from baseline to the end of pulmonary rehabilitation according to history of vascular events. The *p*-value is for non-parametric comparison.

Significantly higher changes in FMD were documented in convalescent COVID-19 patients without a history of vascular events as compared to those with (+2.04% ± 2.30 vs. +0.61% ± 1.83, *p* = 0.013, Figure 1), whereas no difference was found in FMD changes when stratifying patients according to the presence/absence of any CV risk factor (+ 1.91% ± 1.97 vs. +1.71% ± 2.39, *p* = 0.625) and according to the other major clinical and demographic characteristics (Supplemental Table S2).

**Figure 2:**
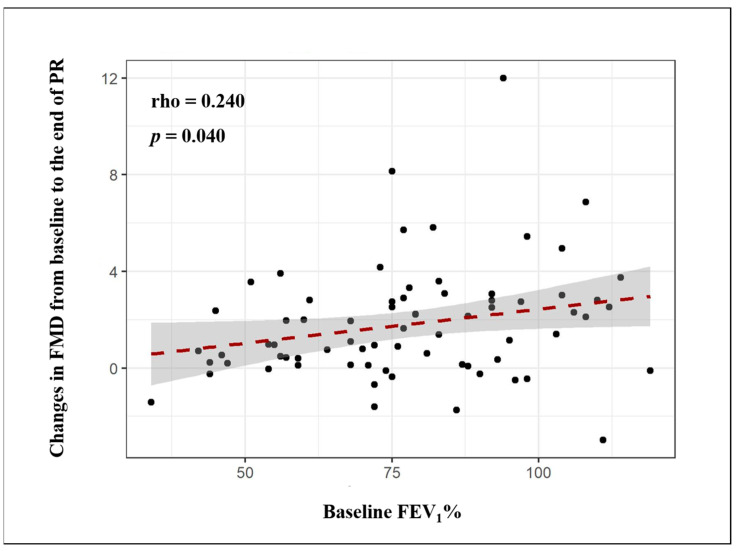
Relationship between changes in flow-mediated dilation (FMD) after pulmonary rehabilitation and baseline values of forced expiratory volume in 1 s (FEV_1_).

Baseline FEV_1_% (rho = 0.372, *p* = 0.001), FVC% (rho = 0.336, *p* = 0.004) and DLCO% values (rho = 0.329, *p* = 0.009) significantly and directly correlated with baseline FMD. Similarly, FEV_1_% (rho = 0.543, *p* < 0.001), FVC% (rho = 0.433, *p* < 0.001) and DLCO% (rho = 0.238, *p* = 0.049) exhibited a direct correlation with FMD values at the end of the PR program. Moreover, baseline FEV_1_% values directly correlated with changes in FMD after PR (Figure 2).

**Figure 3:**
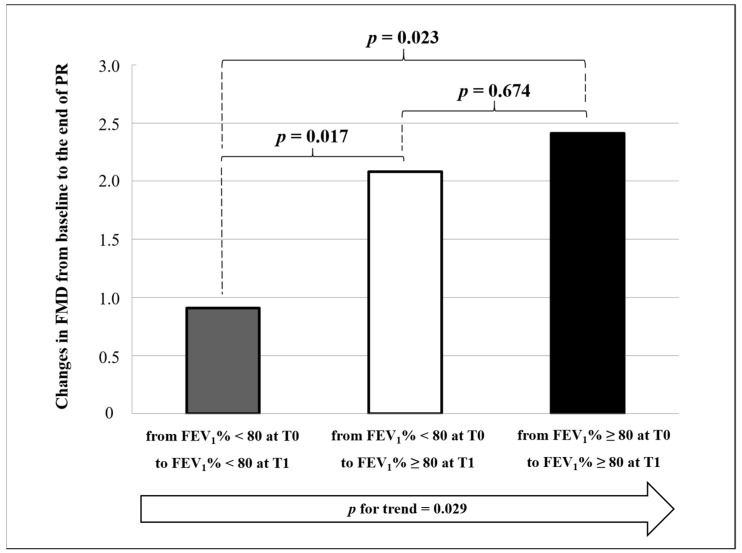
Changes in flow-mediated dilation (FMD) from baseline (T0) to the end of pulmonary rehabilitation (T1) according to forced expiratory volume in 1 s (FEV_1_). All *p*-values are for non-parametric comparisons.

Interestingly, patients with normal FEV_1_% (≥ 80% predicted) during the overall study period or those normalizing FEV_1_% after PR showed a more significant FMD change as compared to patients with persistently impaired FEV_1_% (< 80% predicted) (+2.41% ± 2.78 vs. +2.08% ± 1.87 vs. +0.91% ± 1.75, *p* for trend = 0.029, Figure 3). This finding was confirmed in a multivariate analysis after adjusting for age, gender, history of vascular events, changes in DLCO, changes in FVC, and baseline values of FMD (β = 0.336, *p* = 0.013).

## 4. Discussion

In this study, we documented a significant improvement in clinically evaluated endothelial function of convalescent COVID-19 patients after multidisciplinary rehabilitation, with a ≈71% increase in FMD as compared to baseline values. Moreover, our results consistently show a direct and persistent correlation between the severity of pulmonary and vascular disease, even suggesting that the improvement in endothelial function may be positively correlated with the improvement in pulmonary function.

To date, a growing amount of literature data indicate that SARS-CoV-2 is not limited to the respiratory tract and a multiple organ involvement seems to occur in more severe forms [6]. Thus, in order to adequately manage both the acute phase and the long-term outcomes of COVID-19, a further aspect to consider is the high incidence of CV manifestations [42]. Both arrhythmic and ischemic complications with deranged markers of myocardial injury have been reported among severe COVID-19 patients [11,39,40], with a non-negligible risk of acute cardiac failure and hemodynamic compromise [43,44]. In keeping with this, a very high incidence of stroke and thromboembolic complications has also been reported in more severe forms [45,46]. Moreover, it has been suggested that CV risk may persist beyond the acute phase. This is confirmed by the evidence of echocardiographic and magnetic resonance abnormalities [11,47] as well as the high incidence of arterial and venous events among convalescent COVID-19 patients [48]. However, the mechanisms underlying these acute and post-acute manifestations of COVID-19 are still under investigation [13].

### 4.1. Physiopathology of Endothelial Dysfunction in COVID-19

**Figure 4:**
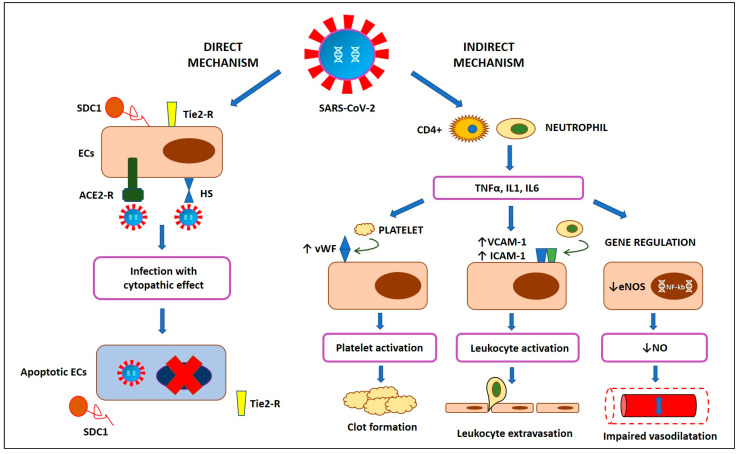
Physiopathology of endothelial dysfunction in coronavirus disease 2019 (COVID-19). SARS-CoV-2: severe acute respiratory syndrome coronavirus 2; ECs: endothelial cells; ACE2-R: angiotensin converting enzyme 2 receptor; HS: heparan sulphate; SDC1: syndecan-1; CD4+: cluster of differentiation 4 cells; TNFα: tumor necrosis factor α; IL1: interleukin 1; IL6: interleukin 6; vWF: von Willebrand factor; VCAM-1: vascular cell adhesion molecule-1; ICAM-1: intercellular adhesion molecule-1; eNOS: endothelial nitric oxide synthase; NF-κB: nuclear factor-κB; NO: nitric oxide.

A growing body of evidence suggests a key role of endothelial dysfunction in the pathogenesis of most COVID-19 manifestations [14], since endothelial cells (ECs) are a preferential target of SARS-CoV-2 with subsequent systemic endotheliitis [15]. It has been shown that SARS-CoV-2 is able to infect cells using the angiotensin converting enzyme 2 (ACE2) receptor, also expressed by ECs [15]. Recently, Stahl et al. detected high plasma levels of Tie-2 receptor and syndecan-1 in critical patients with COVID-19, which reflects the rupture of the endothelial glycocalyx covering the luminal surface of ECs [49]. This is in line with the post-mortem evidence of viral inclusions in apoptotic ECs with microvascular lymphocytic endotheliitis [15]. The disruption of vascular integrity leads to the exposure of the thrombogenic basal lamina and the activation of the clotting cascade [50]. However, endothelial dysfunction is not only a consequence of direct virus infection with subsequent cellular death, potentially depending also on systemic inflammation [50]. Inflammatory cytokines from activated leukocytes, such as interleukin 1 (IL1) and tumor necrosis factor α (TNFα), bind specific receptors on ECs’ surface, thus enhancing the expression of a number of mediators, including intercellular adhesion molecule-1 (ICAM-1), vascular cell adhesion molecule-1 (VCAM-1), E-selectin, P-selectin, fibrinogen and von Willebrand factor (vWF) [51,52]. This results in platelet activation as well as leukocyte adherence and extravasation [52,53,54]. A decline in nitric oxide (NO) bioavailability is another key aspect of a dysfunctional endothelium in COVID-19 [55], potentially depending on the high levels of circulating interleukin 6 (IL6) and other inflammatory markers [56]. A systemic NO deficiency may compromise vascular smooth muscle relaxation [20,57] while reducing the capacity to neutralize oxidative stress and activate NO-mediated antiviral mechanisms (Figure 4) [50].

### 4.2. Clinical Implications

Overall, increasing evidence suggests that endothelial dysfunction may be a key pathogenic mechanism of a systemic prothrombotic state in COVID-19 [50], confirmed by the high incidence of venous and arterial thrombotic complications [42,46]. In keeping with this, the association of microthrombus formation with organ dysfunction and acute respiratory distress has been recently proposed [58,59]. Moreover, the evidence of a persistent endothelial dysfunction after the acute phase due to residual activation of the immune system supports the hypothesis that this mechanism may underline, at least in part, the manifestations of post-COVID-19 syndrome [16]. This has led the European Society of Cardiology to recommend clinical assessment of endothelial function in the follow-up of all convalescent COVID-19 patients for early detection of long-term CV complications [17].

In our cohort study, FMD assessment was used to monitor changes in endothelial function of convalescent COVID-19 patients during multidisciplinary rehabilitation. The importance of rehabilitation in this clinical setting has been discussed in several reports, due to the persistence of a post-COVID-19 syndrome significantly impacting the quality of life and healthcare needs [6,60]. Our finding of a significant and consistent improvement in most pulmonary function tests and exercise capacity substantially confirms the potential beneficial role of PR after the acute phase. In addition to the restoration of functional abilities, PR also aims at a monitoring activity to allow the early identification and management of complications and late-onset manifestations. In this regard, the ≈2% absolute increase in FMD values documented after PR suggests a potential positive impact on endothelial function and CV risk. The clinical significance of our findings is best appreciated when we consider that each 1% absolute increase in FMD is associated with a 12% to 13% decrease in CV events [18,61]. Our result of significantly higher changes in FMD in patients without a history of vascular events is in line with the observation of a better prognosis in this clinical setting [62].

Despite the absence of a control group due to the challenges posed by the pandemic, it can be argued that the baseline FMD values documented in our study population were generally low [21] and directly correlated with FEV_1_%, FVC% and DLCO% values. This direct correlation between the severity of pulmonary and vascular diseases was also confirmed at the end of PR. Moreover, when specifically considering FEV_1_, our results suggest that the improvement in endothelial function could be positively correlated with the improvement in pulmonary function. FEV_1_ is a key spirometry parameter, potentially reflecting either an obstructive or a restrictive pattern. Considering that mean FEV_1_/FVC values were generally normal during the overall study period, the hypothesis of a direct correlation between the restrictive pattern and clinically evaluated endothelial dysfunction can be made in our post-COVID-19 population. In line with our results, a direct correlation between FMD and FEV_1_ has been documented also in other clinical settings [63].

The finding of no significant change in the other parameters of vascular reactivity together with the observation of a significant increase in normalized FMD may be interpreted as a further confirmation that ECs’ integrity and NO bioavailability are the key effectors of the vascular response in our study population. In other words, if the shear stress stimulus is unchanged after PR, we can hypothesize that only the response of ECs has improved. This is consistent with the results of a small previous study, showing that COVID-19 patients exhibit significantly lower FMD values with no significant difference in other parameters of vascular reactivity (e.g., SR_AUC_) when compared to healthy controls [41].

Overall, our findings are in line with previous evidence in other respiratory diseases, supporting the hypothesis that exercise-based PR may have beneficial effects not only on pulmonary function and the quality of life but also on the CV risk profile [64]. The increased NO bioavailability may be a key player in the observed beneficial effects of exercise on CV health [65]. Thus, FMD may be a useful biomarker to monitor vascular adaptations following PR in convalescent COVID-19 patients. It is noteworthy that both D-Dimer and CRP levels resulted in being consistently high in our population after PR. Accordingly, although FMD significantly improved, it remained substantially low at the end of the study period. Overall, a residual CV risk even after PR should be taken into account in this clinical setting.

### 4.3. Limitations

Some relevant limitations of the present study should be addressed. First, the lack of a control group must be considered as a major limitation of the present study. This significantly limits the clinical relevance of our finding on the potential usefulness of PR programs in terms of endothelial function recovery and CV risk reduction. Although a non-PR control group would be desirable, a number of issues related to the current global emergency and the organization of the health system in our region during the pandemic period did not allow it. However, the before–after observational design made it possible to overcome (at least in part) the intrinsic inter-individual heterogeneity, with each participant representing the control of themself.

Moreover, we should consider that FMD assessment is an operator-dependent procedure. In an effort to overcome this limitation, we evaluated the reproducibility of the FMD protocol in advance on a characteristic sample set. Furthermore, to diminish operator-dependent variability and to increase reproducibility, a software approved by the Food and Drug Administration (FDA) was used for our study, permitting an automatic and real-time calculation of the vascular reactivity parameters.

Differences in BAD may represent a further confounding element, potentially affecting FMD values [66]. Interestingly, no significant change in BAD was documented in our study population during the overall study period, thus suggesting that the reported FMD changes are likely due to changes in vascular reactivity.

Finally, we have to consider that our study population consisted of patients with a relatively high prevalence of concomitant CV risk factors, potentially affecting vascular reactivity [37]. To evaluate sources of heterogeneity, we performed a sensitivity analysis after stratifying patients according to the presence/absence of any traditional CV risk factor. Of interest, no significant difference was found between the two different study groups, thus partially offsetting the above limitation.

## 5. Conclusions

Our results suggest a potential role of PR in improving the endothelial function and CV risk of convalescent COVID-19 patients. Such improvement in a clinical measure of endothelial function may be positively correlated with the improvement in pulmonary function. Larger controlled studies are needed to confirm our preliminary findings. This study also supports the potential utility of periodic endothelial function testing in the follow-up of convalescent COVID-19 patients. This could help establish combined and more specific prevention and therapeutic strategies aimed at reducing the long-term CV risk of these patients.

## Figures and Tables

**Table 1:** Baseline characteristics of convalescent COVID-19 patients and changes in body composition, pulmonary function tests, laboratory parameters and other outcome measures after pulmonary rehabilitation (PR).

Variable	Baseline	After PR	*p*-Value
	82	82	
Demographic			
Age, Years	60.4 ± 10.4	-	-
Male Gender (%)	85.4	-	-
Active Smokers (%)	9.8	-	-
History of Smoking (%)	41.5	-	-
Pack-Years	20.4 ± 50.6	-	-
Anthropometric			
Weight (kg)	85.2 ± 18.5	83.7 ± 17.3	0.069
BMI (kg/m^2^)	28.9 ± 5.7	28.4 ± 5.1	0.081
Acute phase COVID-19			
WHO Class III, Severe (%)	35.4	-	-
WHO Class IV, Critical (%)	64.6	-	-
Hospitalization (%)	68.3	-	-
Length of Stay for Hospitalized Patients (days)	23.2 ± 10.5	-	-
High-Flow O_2_ (%)	26.8	-	-
Mechanical Ventilation (%)	23.2	-	-
Lung function			
FEV_1_ (L)	2.4 ± 0.7	2.6 ± 0.7	**<0.001**
FEV_1_% (% predicted)	77.7 ± 20.3	84.5 ± 18.2	**<0.001**
FVC (L)	2.9 ± 0.8	3.2 ± 0.8	**<0.001**
FVC% (% predicted)	74.9 ± 18.9	82.1 ± 16.4	**<0.001**
FEV_1_/FVC	82.4 ± 8.7	81.6 ± 9.5	0.241
DLCO (ml/min/mmHg)	12.9 ± 6.9	12.6 ± 8.2	0.094
DLCO% (% predicted)	57.6 ± 20.0	64.2 ± 20.7	**<0.001**
DLCO/VA (ml/min/mmHg/L)	2.9 ± 1.3	2.7 ± 1.4	0.399
DLCO/VA% (% predicted)	85.2 ± 18.6	89.7 ± 15.0	**0.004**
Arterial blood gas test			
PaO_2_ (mmHg)	75.0 ± 14.2	82.4 ± 12.9	**<0.001**
PaCO_2_ (mmHg)	35.6 ± 3.8	37.0 ± 2.9	**<0.001**
pH	7.44 ± 0.04	7.43 ± 0.04	0.070
HCO_3_ (mEq/L)	25.2 ± 2.6	25.2 ± 2.9	0.974
SpO_2_ (%)	94.5 ± 3.4	96.1 ± 2.7	**<0.001**
Blood laboratory parameters			
Hemoglobin (g/dL)	12.9 ± 1.7	12.6 ± 1.7	**0.032**
Red Cells (10^6^/mL)	4.6 ± 0.6	4.5 ± 0.7	0.104
Hematocrit (%)	39.9 ± 7.2	38.6 ± 4.9	0.080
Leukocytes (10^3^/mL)	8.2 ± 3.4	7.5 ± 2.9	0.059
Platelets (10^3^/mL)	215.7 ± 85.3	199.2 ± 60.3	0.066
Glucose (mg/dL)	95.1 ± 30.0	82.2 ± 15.6	**0.001**
Creatinine (mg/dL)	0.83 ± 0.18	0.85 ± 0.20	0.168
Total Cholesterol (mg/dL)	185.6 ± 41.9	176.3 ± 41.3	0.053
Triglycerides (mg/dL)	160.2 ± 75.4	148.3 ± 54.9	0.146
BUN (mg/dL)	39.4 ± 13.4	33.7 ± 9.7	**0.033**
Uric Acid (mg/dL)	5.1 ± 1.7	5.6 ± 1.5	**0.009**
AST (UI/L)	21.3 ± 11.4	17.2 ± 8.1	**0.001**
ALT (UI/L)	51.7 ± 49.1	33.6 ± 26.9	**<0.001**
CRP (mg/L)	9.9 ± 18.1	9.6 ± 27.2	0.717
D-Dimer (ng/mL)	703.8 ± 651.8	734.1 ± 1694.1	0.788
Blood pressure			
SBP (mmHg)	125.4 ± 12.7	124.1 ± 8.8	0.149
DBP (mmHg)	78.7 ± 8.6	75.2 ± 5.4	**0.009**
Self-assessment scores			
CAT	26.8 ± 3.3	8.7 ± 4.3	**<0.001**
Barthel Index	76.6 ± 23.5	96.5 ± 9.1	**<0.001**
Exercise capacity			
6MWD (meters)	194.9 ± 115.0	343.5 ± 108.3	**<0.001**
Comorbidities			
Hypertension (%)	46.3	-	-
Hypercholesterolemia (%)	6.1	-	-
Hypertriglyceridemia (%)	3.7	-	-
Diabetes Mellitus (%)	17.1	-	-
IFG (%)	1.2	-	-
Obesity (%)	30.5	-	-
Heart Failure (%)	6.1	-	-
Peripheral Artery Disease (%)	1.2	-	-
Atrial Fibrillation (%)	3.7	-	-
History of Myocardial Infarction (%)	15.9	-	-
History of Stroke (%)	2.4	-	-
History of Venous Thromboembolism (%)	0	-	-
History of Malignancy (%)	6.1	-	-
Chronic Kidney Disease (%)	1.2	-	-
COPD (%)	7.3	-	-

BMI: body mass index; WHO: World Health Organization; O_2_: oxygen; FEV_1_: forced expiratory volume in 1 s; FVC: forced vital capacity; DLCO: diffusion lung of carbon monoxide; AV: alveolar volume; PaO_2_: arterial oxygen tension; PaCO_2_: arterial carbon dioxide tension; pH: power of hydrogen; HCO_3_^−^: bicarbonate concentration; SpO_2_: peripheral oxygen saturation; BUN: blood urea nitrogen; AST: aspartate aminotransferase; ALT: alanine aminotransferase; CRP: C-reactive protein; SBP: systolic blood pressure; DBP: diastolic blood pressure; CAT: COPD Assessment Test; 6MWD: six-minute walk distance; IFG: impaired fasting glucose; COPD: chronic obstructive pulmonary disease. Data are presented as mean ± standard deviation. Bold values denote statistical significance (*p* < 0.05).

## Data Availability

The data supporting the findings of this study are available from the corresponding authors upon reasonable request.

## Supplementary Materials

The following are available online at https://www.mdpi.com/article/10.3390/biomedicines9060614/s1, Supplemental Figure S1: Flow chart of study participants, Supplemental Table S1: Pharmacological therapies and oxygen supplementation in convalescent COVID-19 patients undergoing pulmonary rehabilitation, Supplemental Table S2: Impact of major clinical and demographic characteristics on changes in flow-mediated dilation (ΔFMD) in convalescent COVID-19 patients undergoing pulmonary rehabilitation.
