# Supplementary material for: Clinical Assessment of Endothelial Function in Convalescent COVID-19 Patients Undergoing Multidisciplinary Pulmonary Rehabilitation
Source: Biomedicines. 2021 May 28;9(6):614. doi: 10.3390/biomedicines9060614 (PMC8226503; doi:10.3390/biomedicines9060614)
Supplement: Supplementary file 1 [file biomedicines-09-00614-s001.zip › biomedicines-1204569-supplementary.pdf]

## Supplementary online material

# Clinical Assessment of Endothelial Function in Convalescent COVID-19 patients Undergoing Multidisciplinary Pulmonary Rehabilitation.

Pasquale Ambrosino, **MD PhD**[1]* Antonio Molino, **MD**[2]*

Ilenia Calcaterra, **MD**[3] Roberto Formisano, **MD**[1] Silvia Stufano, **PT**[1]

Giorgio Alfredo Spedicato, **PhD FCAS FSA CSPA CStat**[4] Andrea Motta, **PhD**[5]

Antimo Papa, **MD**[1] Matteo Nicola Dario Di Minno, **MD PhD**[6]** Mauro Manisclaco, **MD PhD**[1]**

*The two Authors equally contributed to the manuscript.

**The two Authors share co-seniorship.

## Table of contents


| | |
|---|---|
| **Supplemental Figure S1** | **Flow chart of study participants.** |
| **Supplemental Table S1** | **Pharmacological therapies and oxygen supplementation in convalescent COVID-19 patients undergoing pulmonary rehabilitation.** |
| **Supplemental Table S2** | **Impact of major clinical and demographic characteristics on changes in flow-mediated dilation (ΔFMD) in convalescent COVID-19 patients undergoing pulmonary rehabilitation.** |

**Supplementary Figure S1. Flow chart of study participants.**

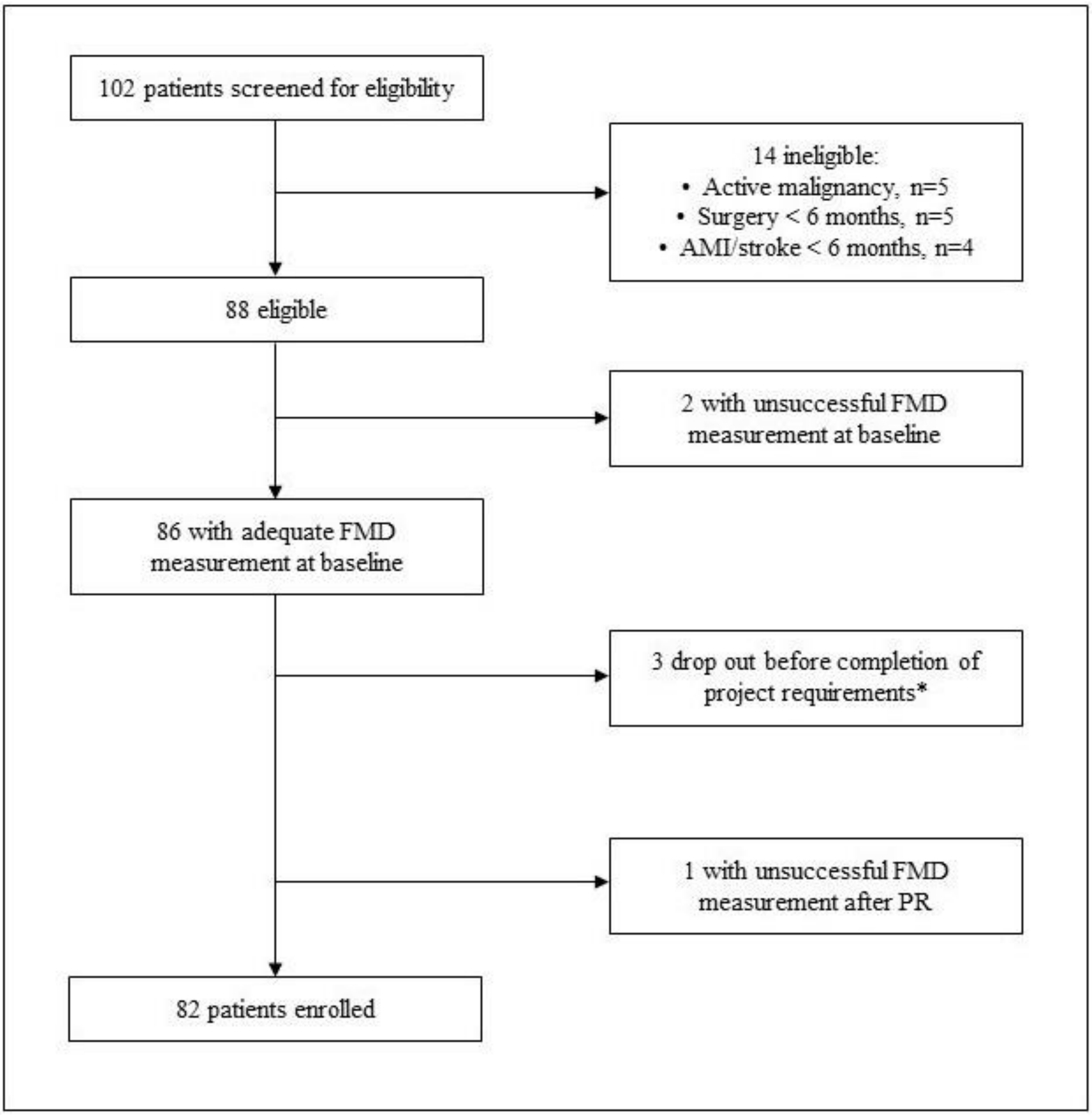


**AMI: acute myocardial infarction; FMD: flow-mediated dilation; PR; pulmonary rehabilitation.**

*2 patients withdrawn informed consent for personal reasons, 1 patient transferred for clinical reasons.

**Supplemental Table S1. Pharmacological therapies and oxygen supplementation in convalescent COVID-19 patients undergoing pulmonary rehabilitation.**

| | **Baseline N=82** | **After PR N=82** | **P values** |
|---|---|---|---|
| Statins (%) | 29.3 | - | - |
| Oral Antidiabetics (%) | 15.8 | - | - |
| Insulin (%) | 12.2 | - | - |
| Allopurinol (%) | 4.9 | - | - |
| β-blockers (%) | 23.2 | - | - |
| ACE-I (%) | 25.6 | - | - |
| CCB (%) | 8.5 | - | - |
| ARB (%) | 8.5 | - | - |
| Diuretics (%) | 15.8 | - | - |
| Antiplatelet Drugs (%) | 22.0 | - | - |
| Oral Anticoagulants (%) | 17.1 | - | - |
| NSAIDs (%) | 23.2 | - | - |
| Corticosteroids (%) | 62.2 | 56.1 | 0.525 |
| Antibiotics (%) | 22.0 | 3.7 | **0.001** |
| LMWH (%) | 54.9 | 23.2 | **<0.001** |
| $O_2$ at Rest (%) | 31.7 | 12.2 | **0.004** |
| $O_2$ Overnight (%) | 26.8 | 12.2 | **0.029** |
| $O_2$ During Exercise (%) | 30.5 | 12.2 | **0.007** |

**N: number; PR: pulmonary rehabilitation; ACE-I: angiotensin-converting enzyme inhibitors; CCB: calcium channel blockers; ARB: angiotensin II receptor blockers; NSAIDs: non-steroidal anti-inflammatory drugs; LMWH: low molecular weight heparin; $O_2$: oxygen.**

**Supplemental Table S2. Impact of major clinical and demographic characteristics on changes in flow-mediated dilation (ΔFMD) in convalescent COVID-19 patients undergoing pulmonary rehabilitation.**

| Independent variable | ΔFMD (presence *vs.* absence) | P value |
|---|---|---|
| Elderly (≥ 65 years) | +2.20% ± 2.76 *vs*. +1.49% ± 1.91 | 0.174 |
| Female gender | +1.84% ± 3.63 *vs*. +1.74% ± 1.99 | 0.899 |
| Mechanical ventilation | +1.89% ± 1.57 *vs*. +1.72% ± 2.46 | 0.779 |
| High-flow $O_2$ | +1.46% ± 1.80 *vs*. +1.87% ± 2.43 | 0.475 |
| WHO class IV | +1.91% ± 2.51 *vs*. +1.49% ± 1.78 | 0.428 |
| Hypertension | +1.65% ± 2.68 *vs*. +1.86% ± 1.89 | 0.682 |
| Hypercholesterolemia | +2.54% ± 2.19 *vs*. +1.71% ± 2.29 | 0.434 |
| Hypertriglyceridemia | +2.89% ± 2.85 *vs*. +1.71% ± 2.25 | 0.383 |
| Diabetes mellitus | +2.29% ± 2.44 *vs*. +1.65% ± 2.25 | 0.335 |
| Smoking | +1.47% ± 2.11 *vs*. +1.79% ± 2.31 | 0.710 |
| Obesity | +1.24% ± 1.66 *vs*. +1.99% ± 2.48 | 0.171 |
| Heart failure | +2.23% ± 3.21 *vs*. +1.73% ± 2.23 | 0.637 |
| COPD | +1.33% ± 2.51 *vs*. +1.79% ± 2.27 | 0.634 |
| History of malignancy | +0.77% ± 1.77 *vs*. +1.82% ± 2.30 | 0.321 |

**WHO: world health organization; $O_2$: oxygen; COPD: chronic obstructive pulmonary disease.**
